# Finsterer- Bancroft-Plenk operation and Bouveret's syndrome: a rare association

**DOI:** 10.1590/0102-67202025000060e1929

**Published:** 2026-04-10

**Authors:** Philipe Franco do Amaral Tafner, Giulia Trucolo de Brito, Joao Guilherme Oliveira Vaz, Bruna Mattos Vanetti de Albuquerque, Ana Carolina Arantes, Ary Augusto de Castro Macedo, Valdir Tercioti, João de Souza Coelho, Nelson Adami Andreollo, Luiz Roberto Lopes

**Affiliations:** 1 Universidade Estadual de Campinas, Faculdade de Ciências Médicas, Department of Surgery and Gastrocentro, Digestive Diseases Surgical Unit – Campinas (SP), Brazil.

**Keywords:** Duodenal Ulcer, Cholelithiasis, Intestinal Fistula, Gastrectomy, Úlcera Duodenal, Colelitíase, Fístula Intestinal, Gastrectomia

## Abstract

Peptic ulcer disease (PUD) presents different spectrums of evolution and severity. Epigastric pain is the patient's most important complaint and may be associated with other complications, such as bleeding, perforations and stenosis, and comorbidities. Currently, the incidence of evolution to severe forms and complications of PUD has decreased significantly, mainly due to the indiscriminate use of proton pump inhibitors. The authors communicate an uncommon situation of a 36-year-old male patient with PUD and duodenal stenosis associated with complicated cholelithiasis. After releasing the adhesions with the liver, a cholecystoduodenal fistula was identified, and the presence of a large gallstone impacted in the duodenum (1.2 cm), characterized as Bouveret syndrome. The Finsterer-Bancroft-Plenk technique was chosen, with preservation and mucosectomy of the antrum, closure of the duodenum, followed by partial gastrectomy with Roux-en-Y reconstruction, truncal vagotomy, cholecystectomy and duodenal suture correcting the cholecystoduodenal fistula.

## ARTICLE HIGHLIGHTS

- Peptic ulcer disease (PUD) presents different spectrums of evolution and severity. Epigastric pain is the patient's most important complaint and may be associated with other complications, such as bleeding, perforations and stenosis, associated with comorbidities.
- Bouveret syndrome is a rare syndrome with nonspecific symptoms and prolonged evolution, characterizing duodenal obstruction by a large gallstone migrated through a cholecystoduodenal fistula.
- It is currently known as the Finsterer-Bancroft-Plenk technique, the preservation of the antrum and removal of the antral mucosa associated by partial gastrectomy.
- The Finsterer-Bancroft-Plenk technique is still a surgical option in the face of complex duodenal stenoses secondary to PUD.

## CENTRAL MESSAGE

Peptic ulcer disease (PUD) presents different spectrums of evolution and severity. Epigastric pain is the patient's most important complaint and may be associated with other complications, such as bleeding, perforations and stenosis, associated with comorbidities. The association of duodenal stenosis, complicated cholelithiasis, cholecystoduodenal fistula and a large biliar calculous (1.2 cm) is an uncommum situation. The Finsterer-Bancroft-Plenk technique was chosen, with preservation and mucosectomy of the antrum, closure of the duodenum, followed by partial gastrectomy with Roux-en-Y reconstruction, truncal vagotomy, cholecystectomy and duodenal suture correcting the cholecystoduodenal fistula.

## PERSPECTIVES

The Finsterer-Bancroft-Plenk technique is still a surgical option in the face of complex duodenal stenoses secondary to PUD, even with recent technological innovations in medicine and digestive surgery. However, knowledge of history, the study of both surgical techniques and evolutionary processes cannot be ignored, in addition to the surgeon's experience and constant improvement, which allow the resolution of difficult cases.

## Graphical abstract

**Figure:**
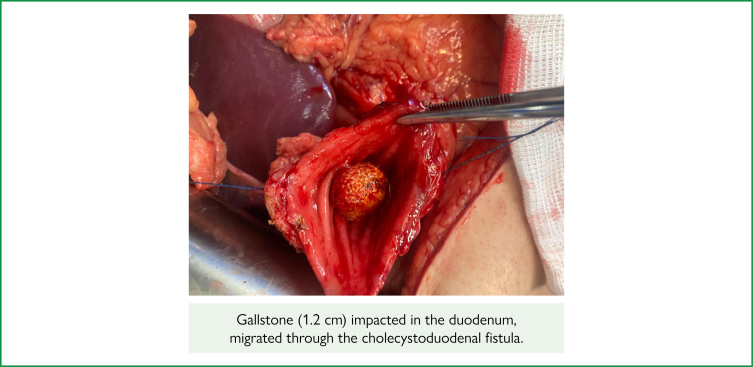

## RESUMO

A doença ulcerosa péptica (DUP) apresenta diferentes espectros de evolução e gravidade. A dor epigástrica é a queixa mais importante do paciente e pode estar associada a outras complicações, como hemorragias, perfurações e estenose, além de comorbidades. Atualmente, a incidência de evolução para formas graves e complicações da DUP diminuiu significativamente, principalmente devido ao uso indiscriminado de inibidores da bomba de prótons. Os autores relatam um caso incomum de um paciente do sexo masculino, de 36 anos, com DUP e estenose duodenal associada à colelitíase complicada. Após a liberação das aderências hepáticas, foi identificada uma fístula colecistoduodenal e a presença de um grande cálculo biliar impactado no duodeno (1,2 cm), caracterizado como síndrome de Bouveret. A técnica de Finsterer-Bancroft-Plenk foi escolhida, com preservação e mucosectomia do antro, fechamento do duodeno, seguida de gastrectomia parcial com reconstrução em Y de Roux, vagotomia troncular, colecistectomia e sutura duodenal para correção da fístula colecistoduodenal.

## INTRODUCTION

Peptic ulcer disease (PUD) presents different spectrums of evolution and severity. Epigastric pain is the patient's most important complaint and may be associated with other complications, such as bleeding, perforations and stenosis, associated with comorbidities. Therefore, the differential diagnosis, despite the severity, includes cholelithiasis and its complications, in addition to gastroesophageal reflux disease and even neoplasms^4,6,9^. Currently, the incidence of evolution to severe forms and complications of PUD has decreased significantly, mainly due to the indiscriminate use of proton pump inhibitors. The treatment of PUD, therefore, has migrated in recent decades from the operating room to outpatient clinics and pharmacy counters. Perforations and stenoses, however, are still diagnosed, with surgical treatment being indicated, and may be related to irregular use of these medications as well as inadequate medical follow-up^9^.

The objective is to communicate an uncommon situation of PUD with stenosis associated with complicated cholelithiasis.

## PATIENT’S RECORDS

**Figure 1:**
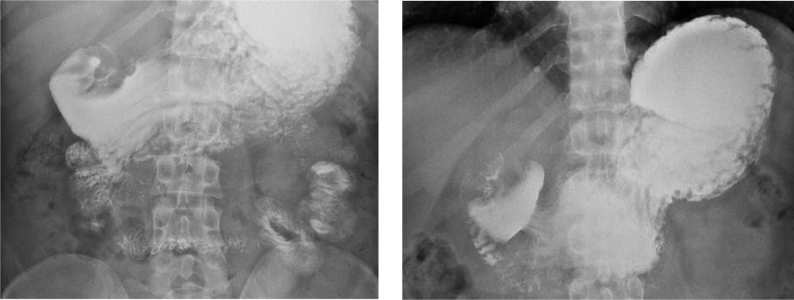
Abdominal X-rays after barium ingestion, showing duodenal obstruction (left and right).

**Figure 2:**
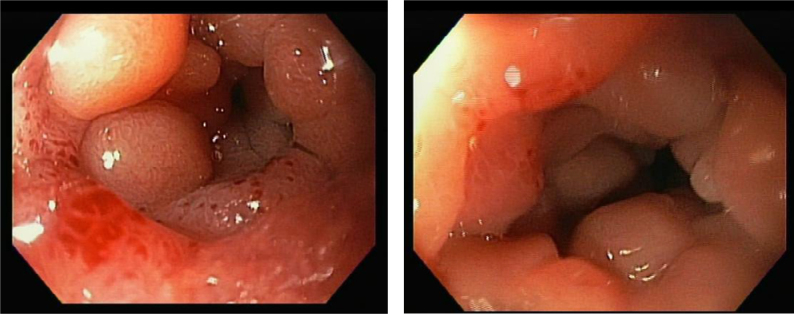
Upper digestive endoscopy showing duodenal bulb stenosis, without other lesions (left and right).

**Figure 3:**
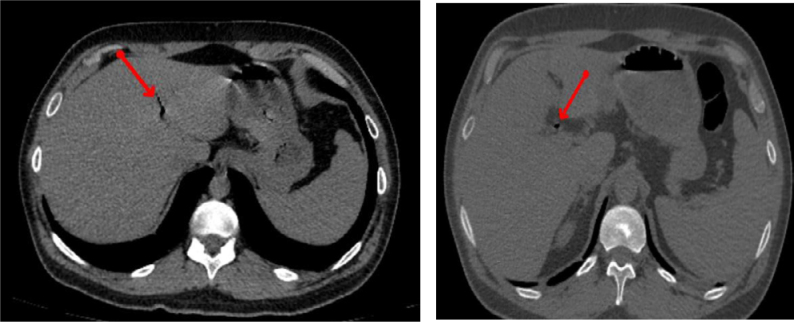
Abdominal computed tomography scans showing aerobilia (arrows — left and right).

**Figure 4:**
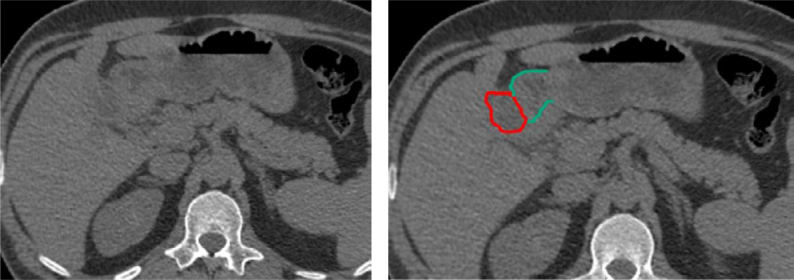
Abdominal computed tomography scans (without intravenous contrast) showing thickening of the duodenal wall and gallbladder, with a probable fistulous tract (left and right).

**Figure 5:**
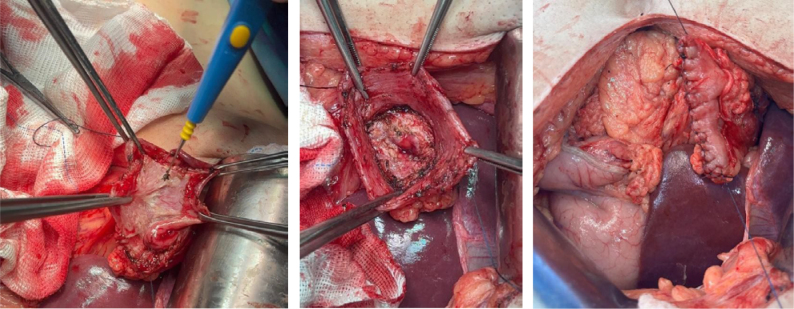
The Finsterer-Bancroft-Plenk technique, with preservation of the antrum, mucosectomy (left), duodenal closure (middle), and suturing of the remaining seromuscular wall (right).

**Figure 6:**
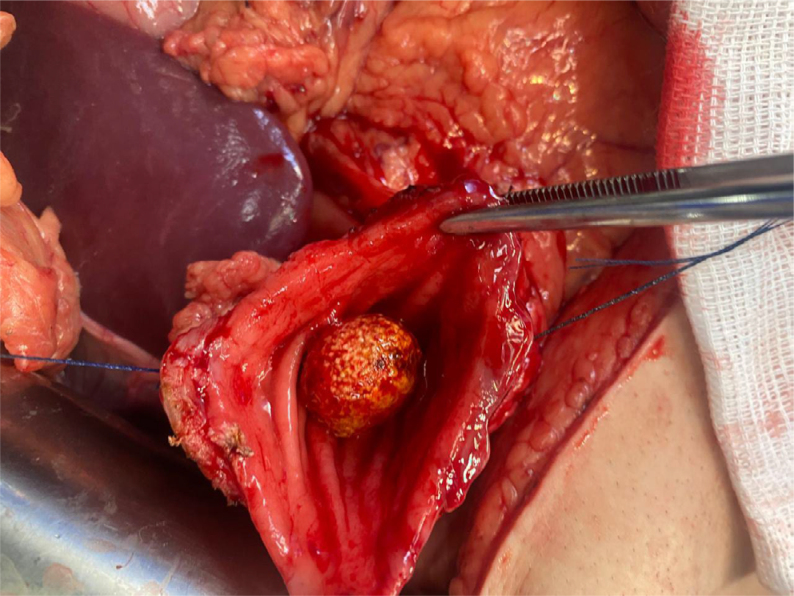
Gallstone (1.2 cm) impacted in the duodenum, migrated through the cholecystoduodenal fistula.

A 36-year-old male patient was evaluated at the outpatient clinic for suspected pyloric stenosis of peptic etiology. He presented with complaints of long-standing epigastric pain and heartburn with progressive worsening of symptoms for two years. He reported difficulty ingesting solid foods, with preference for liquids, having presented on more than one occasion episodes of vomiting content similar to gallstones, in addition to weight loss of 30 kg during the period. Furthermore, he had symptoms of symptomatic cholelithiasis without effective resolution or adequate medical follow-up. He had already presented choledocholithiasis requiring endoscopic retrograde cholangiopancreatography for stone removal. He reported different pain compared to the biliary lithiasis condition and worsening intensity. There were no other comorbidities, despite smoking and social alcohol consumption. At the outpatient consultation, contrast radiographs of the esophagus, stomach, and duodenum showed duodenal stenosis with gastric stasis and an image with contrast accumulation in the region near duodenal obstruction (Figure 1). Upper digestive endoscopy showed grade A esophagitis according to Los Angeles classification and stenosis in the duodenal bulb, suspected possible peptic stenosis (Figure 2). Abdominal ultrasound showed thickening of the gallbladder wall and cholelithiasis. Preoperative computed tomography demonstrated signs of aerobilia (Figure 3) and thickening of the duodenal bulb and gallbladder, of nonspecific etiology (Figure 4). The patient underwent exploratory laparotomy, which revealed an intense inflammatory reaction with adhesions between the gallbladder, stomach, and duodenum. After releasing the adhesions with the liver, a cholecystoduodenal fistula was identified, as well as the presence of a large gallstone impacted in the duodenum (1.2 cm), characterized as Bouveret syndrome^5^ (Figure 5). Cholecystectomy and duodenal suture were performed using a non-absorbable suture, thus correcting the cholecystoduodenal fistula. Due to significant inflammation in the duodenum, proximity to the common bile duct and therefore high risk of inadvertent bile duct injury, in addition to risk of duodenal stump fistula, an approach similar to duodenal ulcers with difficult duodenal stump was chosen. The Finsterer-Bancroft-Plenk technique^2,7,8,10,11^ was chosen, with preservation and mucosectomy of the antrum and closure of the duodenum, followed by partial gastrectomy with Roux-en-Y reconstruction and truncal vagotomy (Figures 6). A penrose drain was left in the right lateral region of the abdomen.

The patient presented low-output biliary fistula in the postoperative period, secondary to duodenal suture of the cholecystoduodenal fistula, but with satisfactory evolution. He was discharged on the ninth postoperative day and the fistula closed in two weeks.

## DISCUSSION

Before the proton inhibitor era, some technical options for gastric and duodenal resection were employed for surgical treatment of peptic ulcer disease and its complications. Currently, these technical options are not necessary, and partial gastrectomy with duodenal closure and Roux-en-Y reconstruction is the most commonly employed technique^5,9^.

However, in some unusual situations, the experience and knowledge of these old and widely used techniques in the past are good options to avoid complications and negative outcomes. In this case, Bouveret syndrome^5^, in addition to being a rare syndrome with nonspecific symptoms and prolonged evolution, characterizing duodenal obstruction by a large gallstone migrated through a cholecystoduodenal fistula, was not diagnosed preoperatively with complementary exams, and was only confirmed during exploratory laparotomy. Due to the previous diagnosis of peptic ulcer disease with duodenal stenosis being more likely, the patient was referred to the operating room with the intention of treating this complication. Although it was not the main hypothesis, Bouveret syndrome was confirmed upon verifying the presence of a cholecystoduodenal fistula with stones in the duodenal bulb^1,3,5^.

Due to the intense inflammatory process from probable previous peptic ulcer in the duodenum and previous acute cholecystitis, multiple adhesions were found in the liver and large intestine that were difficult to manipulate^1,3^. The patient's previous history of having undergone endoscopic retrograde cholangiopancreatography at another service confirms that he had cholelithiasis.

The surgical technique employed was initially described by van Eiselberg for treatment of complex duodenal stumps, a situation that was frequently encountered in duodenal ulcers, described as penetrating ulcers, near the common bile duct. van Eiselberg indicated gastric transection at the antrum level without organ resection and reconstruction of transit with the proximal portion of the stomach. In 1918 the technique was improved by Finsterer and Plenk and later by Bancroft, who finally proposed a similar technique with details for closing the gastric antrum^2,4,7,8,10,11^.

Therefore, it is currently known as the Finsterer-Bancroft-Plenk technique, the preservation of the antrum and removal of the antral mucosa associated with partial gastrectomy. After mucosectomy in the antrum, the pylorus is closed, followed by suture to collapse the remaining seromuscular wall of the antrum. Mucosectomy is essential, mainly for removal of antral gastrin-producing cells, preventing secondary hyperchlorhydria and exacerbated release of gastrin into the bloodstream^2,4,7,8,10,11^.

The Finsterer-Bancroft-Plenk technique, employed by other surgeons in the past, was classically described for treatment of duodenal peptic ulcer disease with difficult duodenal stump approach, avoiding manipulation of the region. Risky manipulation of this topography with an intense inflammatory process increases the probability of inadvertent bile duct injury and other structures near the duodenum, such as the hepatic hilum. Resection of the duodenum and gallbladder is imprudent in most situations similar to this clinical case. In the consulted literature, rare cases of Bouveret syndrome are described, but there are other, even rarer reports with the evolution of a cholecystoduodenal fistula and post-pyloric obstruction by stones secondary to PUD in the duodenum. In this clinical case, both calculous cholecystopathy and associated PUD contributed to the symptoms and clinical evolution of the patient^1,3,5^.

## CONCLUSIONS

The Finsterer-Bancroft-Plenk technique is still a surgical option in the face of complex duodenal stenoses secondary to PUD. Development and technological innovation are part of medicine and digestive surgery. However, knowledge of history, the study of both surgical techniques and evolutionary processes cannot be ignored, in addition to the surgeon's experience and constant improvement, which allow the resolution of difficult cases.

## Data Availability

The Informations regarding the investigation, methodology and data analysis of the article are archived under the responsibility of the authors.
